# Randomized Controlled Trial of Adjunctive Social Cognition and Interaction Training, Adjunctive Therapeutic Alliance Focused Therapy, and Treatment As Usual Among Persons With Serious Mental Illness

**DOI:** 10.3389/fpsyt.2019.00364

**Published:** 2019-06-11

**Authors:** Ilanit Hasson-Ohayon, Michal Mashiach-Eizenberg, Adi Lavi-Rotenberg, David Roe

**Affiliations:** ^1^Department of Psychology, Bar-Ilan University, Ramat Gan, Israel; ^2^Department of Health System Management, Max Stern Yezreel Valley College, Emek Yezreel, Israel; ^3^Department of Community Mental Health, Faculty of Social Welfare and Health Sciences, University of Haifa, Haifa, Israel; ^4^Department of Clinical Medicine, Psychiatry, Aalborg University, Aalborg, Denmark

**Keywords:** social cognition, therapeutic alliance, serious mental illness, randomized controlled trial, intervention

## Abstract

As one of the areas of greatest concern for people with serious mental illness (SMI) are unmet social needs, psychosocial interventions have been developed to address them. The current study utilized a randomized controlled trial to examine the effectiveness of social cognition and interaction training (SCIT) versus a therapeutic alliance focused theraphy (TAFT) versus a treatment-as-usual (TAU) control group on social functioning and quality of life as primary outcomes and social cognition variables as secondary outcomes. Sixty-three persons between the ages of 24 and 69 years with SMI (41 men and 22 women), completers of the trial (23 in SCIT, 20 in TAFT, and 20 in TAU), were assessed at baseline, completion, and at a 3-month follow-up with measurements assessing social cognition (The Facial Emotion Identification Task, The Faux pas test, The Ambiguous Intentions Hostility Questionnaire) social functioning, (The Social Skills Performance Assessment, The Wisconsin Social Quality of Life Scale), and therapeutic alliance (adapted version for group of system for observing family therapy alliance). Results reveal that the two interventions were more effective than the control condition (TAU) in reducing attribution bias anger scores, SCIT was also effective in improving theory of mind (as can be seen in Faux pas test scores), and the TAFT in improving emotion recognition and reducing intentionality attribution bias scores. Improvement was related to therapeutic alliance which did not differ between the two intervention groups. Considering the role of alliance, it is recommended to consider the integration of the two studied interventions with other approaches that emphasize alliance and reflection.

**Clinical Trial Registration:** www.ClinicalTrials.gov, identifier NCT02380885.

## Introduction

Serious mental illness (SMI) is associated with impaired social cognition, which in turn hinders quality of life and functioning (1). Social cognition refers to the cognitive processes that underlie social interactions (2, 3) and include elements of emotion identification, theory of mind (ToM), and attributional biases (2). Deficits in social cognition have been shown to be relatively non-responsive to pharmacological interventions (4); in addition, a good social quality of life (SQoL) has been reported by people with SMI to be their greatest unmet need (5). The growing recognition of the devastating impact of social cognition deficits on the lives of people with SMI has generated attempts to develop interventions that target social cognition as a means of improving social functioning and SQoL.

One such intervention is social cognition and interaction training (SCIT) (6). SCIT is a manualized, group-based intervention that has demonstrated efficacy in improving social cognition and social functioning in both inpatient and outpatient samples (7–10). It was developed to address the three core domains of social cognition: emotion perception, ToM, and attributional style. Most studies documenting social cognition deficits were conducted among specific diagnostic groups [e.g., Ref. (1)]. Nonetheless, the Substance Abuse & Mental Health Services Administration (SAMHSA) definition of SMI as presenting a category of disorders with low GAF score and literature, suggesting that diverse diagnosis share similar social deficits (11, 12), justify implementing interventions aimed at improving social deficits across different diagnoses groups (13–16). Indeed, SCIT has been implemented among people with a range of psychiatric diagnoses including schizophrenia bipolar disorder (16), autism spectrum disorders (17), and a diverse group of people with SMI (15).

SCIT emphasizes a set of techniques designed to ameliorate social cognitive deficiencies and biases to promote change. To test the effectiveness of this set of techniques, the current study compared the impact of SCIT to a different intervention, namely, therapeutic alliance focused therapy (TAFT). TAFT is a type of group therapy adapted from a model of observing family therapy alliances (18), which focuses on the group therapeutic alliance. To the best of our knowledge, the only trial to date that assessed the effectiveness of TAFT was conducted with parents of persons with SMI. In that study, a therapeutic alliance therapy was compared with psychoeducational intervention, and no differences between the two conditions were observed in outcome variables (19, 20). It should be noted that in that study both interventions seemed beneficial but without a control group conclusions were limited. In the current study, we also compared both interventions (SCIT and TAFT) with a treatment as usual (TAU) control group.

The comparison between SCIT, which includes training in social cognition, and TAFT, a treatment that emphasizes therapeutic relations factors, and a control group, enables to compare a didactic approach by means of improving social cognition (SCIT) to an experiential approach emphasizing positive group therapeutic alliance (TAFT). Notably, SCIT is thought to improve social functioning *via* improved social cognition; alternatively, there is also evidence suggesting that improvements in psychosis may be achieved *via* generic factors, such as therapeutic alliance (21), which is the focus of TAFT. In fact, therapeutic alliance is regarded as a common factor in psychotherapy, beyond different psychotherapeutic approaches, that is important in improving outcome (22–24). Interestingly, while therapeutic alliance as a common factor may act as a mechanism in SCIT, focusing on improving alliance in TAFT may facilitate social cognition, as it requires self and other awareness regarding interpersonal interactions.

Hypotheses of the current study were as follows: (1) participants who receive either SCIT or TAFT will show greater improvement in SQoL and social functioning than participants who receive TAU [since social functioning and SQoL may be perceived as presenting objective and subjective complementary outcomes (25) we included both]; (2) participants in both SCIT and TAFT will show greater improvement in social cognitive variables (ToM, emotion recognition, attribution errors) than participants who receive TAU; and (3) participants’ improvement will be related to therapeutic alliance. 

## Materials and Methods

### Research Setting

The current randomized controlled trial (RCT) was an intervention study that assessed the effectiveness of SCIT versus TAFT in a psychiatric community setting in Israel (Clinicaltrial.gov ID NCT02380885). Data were collected between the years 2014 and 2018. Previous studies derived from this larger project were conducted on the basis of baseline partial samples of the current study (25, 26). Approval for the study was obtained from the ethics committee of the Department of Psychology at Bar-Ilan University, as well as from two psychiatric hospital committees. After receiving a detailed explanation of the study, all research participants provided written informed consent. Data were collected by an experienced mental health practitioner who was trained to administer.

### Participants

A total of 179 participants, between the ages of 20 and 69 years, participated in the study. Of these participants, 59% were men, and 41% were women. All participants had a case record of SMI and a psychiatric disability of at least 40% (determined by a medical committee, made up in part by a psychiatrist), and met the criteria for National Insurance Institute of Israel (NII) disability benefits (a roughly comparable process to attaining the designation of SMI in the United States). As previous research showed that 86% of 16,000 people in Israel who had a psychiatric disability of at least 40% had a diagnosis of a psychotic-related disorder (27), it is likely that most of the participants in our study sample had a psychotic-related disorder. In addition to having a diagnosis of SMI, inclusion criteria were participants’ fluency in Hebrew and their provision of informed consent. As mentioned in the Introduction, in keeping with the SAMHSA definition of SMI as presenting a category of disorders with a low Global Assessment of Functioning (GAF) score, and the idea that diverse diagnoses share similar social deficits (11, 12), different SMI diagnoses were included (it should be noted that SMI is regarded as including severe forms of depression, and obsessive compulsive disorder, with a predominance of schizophrenia spectrum disorders) (11).

**Figure 1:**
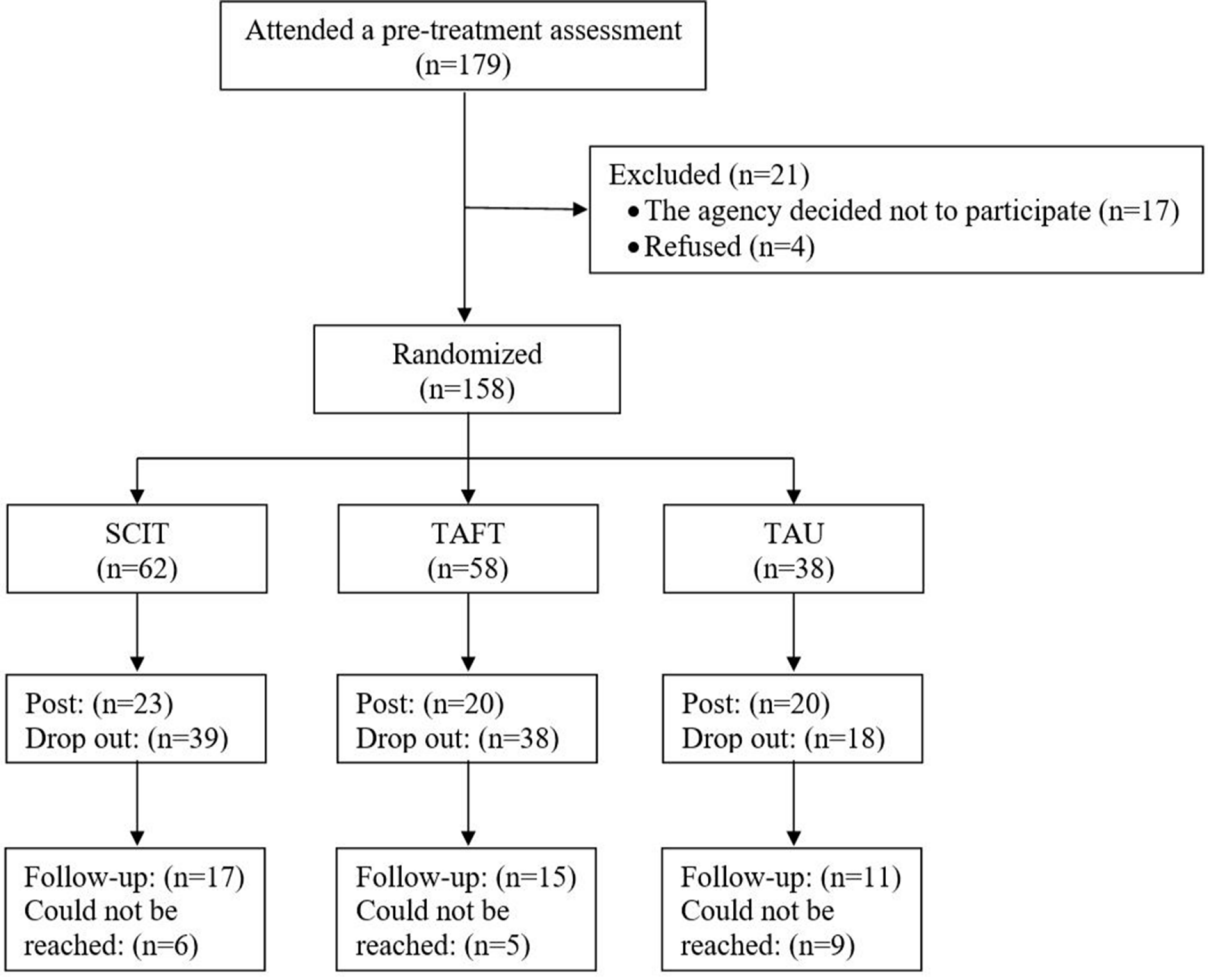
Participant flow chart.

**Figure 1** shows the participants’ flowchart. After the baseline data collection, one of the psychiatric rehabilitation agencies decided not to participate in the study (17 participants). These participants did not take part in randomization. In addition, four participants from another agency also declined to participate in the research. The remaining 158 participants, from four different sites (the number of participants in sites ranged from 20 to 34), were randomized into three groups: 62 to SCIT, 58 to TAFT, and 38 to TAU. Randomization was done *via* lottery after baseline assessments. At certain sites, the number of potential participants was relatively small; a modification of the randomization was therefore made. According to this modification, each participant had the same higher probability to be randomized to one of the intervention groups (as opposed to TAU). This modification ensured sufficiently large SCIT and TAFT groups at these sites.

The completion rate for post-intervention assessments was low: 37% for the SCIT group (n = 23, the number of completers in groups ranged from 2 to 7), 34% for the TAFT group (n = 20, the number of completers in groups ranged from 1 to 7), and 53% for the TAU group (n = 20, the number of participants in groups ranged from 2 to 5). A comparison of the demographic variables between all of the participants (SCIT, TAFT, and TAU) who completed post-intervention assessments, and all of the participants who did not revealed that the group of participants who completed the post-intervention assessments included a larger number of people who worked in a supported employment setting and a lower number of people who worked in a regular work setting, χ^2^(2) = 7.8, *p* < .05. The groups did not, however, differ in gender, marital status, age, or education.

Demographic characteristics of the participants (who completed the post-intervention assessments) are presented in **Table 1**. There were no statistically significant differences between groups (i.e., SCIT, TAFT, and TAU).

**Table 1:** Comparison of baseline characteristics between groups (participants who completed Time 2 assessment).

	SCIT *N* = 23		TAFT *N* = 20		TAU *N* = 20		P^a^
	N	%	N	%	N	%	
Gender: Man Woman	12 11	52% 48%	14 6	70% 30%	15 5	75% 25%	.251
Marital status: Single Married Divorced/Widow	20 1 2	87% 4% 9%	12 3 5	60% 15% 25%	16 1 3	80% 5% 15%	^b^
Occupation: Supported employment Regular work Unemployed	15 3 5	65% 13% 22%	11 3 6	55% 15% 30%	9 2 9	45% 10% 45%	^b^
	*Mean ± SD*		*Mean ± SD*		*Mean ± SD*		
Age (years) min-max	38.9±11.0 25-63		41.4±12.2 24-69		39.7±9.4 24-57		.824
Education (years) min-max	12.5±1.8 8-17		12.0±1.9 8-15		12.3±1.9 8-16		.283

a Differences between the groups were tested with the chi-square test for categorical variables and with the Kruskal–Wallis test for continuous variables.

b For this variable, it is not possible to present the chi-square P value because of the sample size.

### Measures

Scales and tests that were used in this trial were all previously used in their Hebrew version and showed sufficient reliability and validity. See details below.

*The Facial Emotion Identification Task (FEIT)* (28) is a widely used measure of emotion perception and is indexed by the number of correctly identified emotions out of a total of nineteen pictured faces. Emotions include happiness, anger, sadness, fear, surprise, and shame. The FEIT has demonstrated good reliability in studies on schizophrenia (15, 28, 29). In the current study, we examined reliability *via* test–retest reliability among a sub-sample of 25 of the respondents who participated in the reliability test. The correlation between the two measurements was 0.76.

The Hebrew-language version (30) of *the Faux Pas Test* (31) was used in this study to assess TOM. This measure consists of 10 stories in which a faux pas has occurred and 10 stories in which no faux pas has occurred (control stories). A faux pas is considered to have occurred when the speaker says something without taking into account that the listener might not want to hear this story or might be hurt by it. After each story, the participants are asked seven questions regarding their recognition of the occurrence of a faux pas (e.g., their understanding of the mental state of the speaker and listener, or their understanding of the emotional state of the listener). This task assesses emotional and cognitive attributions, and the score for each story ranges between 0 and 7 (task range, 0–70). Cronbach’s alpha in our previous study was high (0.91 in 15), and in the current study, it was found to be satisfactory at 0.73.

The Hebrew-language version (15) of *the Ambiguous Intentions Hostility Questionnaire (AIHQ) *(32) was administered to participants. The AIHQ is a measure of attributional style for situations with negative outcomes and ambiguous causality. Participants are asked to read each of five vignettes, to imagine that the scenario is happening to her or him (e.g., “You walk past a group of teenagers at a mall and you hear them start to laugh”), and to write down the reason why the other person (or persons) acted the way he/she/they did toward the participant. Two independent raters subsequently code this written response on a five-point Likert scale for the purpose of computing a “hostility bias.” The participant then rates the degree to which he or she thinks the other person (or persons) performed the action on purpose, how angry this action would make the participant feel, and how much the participant would blame the other person (or persons). The AIHQ has been shown to have very good levels of reliability and inter-rater agreement [intra-class correlation (ICC) = 0.80+] and to be correlated with other measures of paranoia and hostility (8, 32). Our previous study showed high inter-rater reliability (ICC = 0.85) of the Hebrew version used with SMI (15). In the present study, Cronbach’s alphas were 0.65, 0.69, 0.74, and 0.72 for hostility bias, intentionality, anger, and blame, respectively. It should be noted that an aggression score was not used in the current study because it reflects a behaviorally anticipated response rather than biases in attribution.

*The Social Skills Performance Assessment (SSPA)* (33) was used to assess social functioning. In this task, the subject and the assessor engage in two 3-min role-play conversations (“scenes”) on pre-determined topics (e.g., “Your landlord has not fixed a leak that you told him about last week, and now you are calling him on the phone to follow up”). Role plays are tape-recorded and rated by independent coders. The SSPA has good face validity as a social skills measure, and among individuals with schizophrenia, it has shown excellent inter-rater reliability, good test-retest reliability, and good convergent validity with a measure of activities of daily living (33). Each domain is rated on a five-point Likert scale with higher scores signifying greater social skills. Domains are summed to yield total scores for each scene. Permission to translate and use the scale was obtained from the developers. Cronbach’s alpha in the current study was 0.96. In addition, agreement between raters on the SSPA total score was measured by the mean ICC, with a high and satisfactory mean score of 0.89.

*The Wisconsin* SQoL *Scale* (34). Participants’ SQoL was assessed *via* the social subscale of the Wisconsin Quality of life Index-Mental Health (QLI-MH) that was developed by Becker et al. (34) and translated into Hebrew (35). It consists of 12 self-report items. Five items reflect the individual’s level of satisfaction with his/her social QoL, two items reflect the individual’s number of friends and perceived support, and there are five items that might be used as weights representing the subjective importance of the first five items. For the purposes of the current study, we used only the first five items. The scores range from 0 to 7 with a higher score indicating a higher social QoL. The scale has acceptable reliability and validity in both the English- and Hebrew-language versions (34–36). In the current study, Cronbach’s alpha was 0.80.

*Therapeutic alliance* was measured by an adapted version for group of system for observing family alliance (18), previously used in the context of group therapy for parents of persons with SMI (19). This self-report measure consists of four sub-scales: 1) commitment to the therapeutic process, 2) emotional relationship with the therapist, 3) feeling secure within the therapeutic setting, and 4) a sense of a mutual goal. Cronbach’s alpha of the subscales ranged from 0.42 to 0.70.

### Interventions

Both SCIT and TAFT were provided weekly over a period of 6 months (20–24 sessions). Each session was an hour long. The mental health professionals who provided both the SCIT and TAFT interventions participated in a 1-day workshop and in supervision sessions held once every 2 weeks during the study period. Training was offered to the staff at the rehabilitation centers, and selection of candidates for the training, was based on professional training, experience, and the role they occupied in their respective rehabilitation centers. Training was supervised by the first and last authors (IH-O and DR). Fidelity ratings were done *via* visits to the group after the midway point. These visits included intertwining participants and filling out a scoring sheet by a researcher coordinator [Ref. (6) for SCIT; adaptation of scoring sheet from Ref. (19) for TAFT].

**Social Cognition and Interaction Training** (SCIT) (6) is a manualized group-based intervention that targets the three core social cognitive deficits in schizophrenia: emotion perception, ToM, and attributional style. The SCIT intervention consists of three phases: 1) Emotion Training, 2) Figuring out situations, and 3) Integration. It is delivered by one to two therapists over 20–24 weekly sessions, with each session lasting 60 min, and it involves the use of didactic instruction, videotape and computerized learning tools, and role-play methods to improve social cognition. Techniques include a variety of guessing games and fact-finding exercises, and generating attributions for events in the clients’ lives with an emphasis on possible situational factors that should be considered.

**The TAFT intervention**. The manual of the TAFT intervention is an adaptation of the manual for family focused alliance intervention (18), which we previously used for an intervention for parents of persons with severe mental illness (19, 20). This manual consists of the four elements of the therapeutic alliance according to the family alliance intervention. These elements are 1) commitment to the therapeutic process, 2) emotional relationship with the therapist, 3) feeling secure within the therapeutic setting, and 4) a sense of a mutual goal. The therapist manual consists of detailed guidelines regarding factors that enhance the therapeutic alliance (e.g., sharing the format of the therapy with the group; explaining the rationale behind the group; and asking participants to share their goals, questions, and thoughts about the therapy) as well as factors that may harm it (e.g., pressure clients to participate or failure to detect or address problems might make the therapeutic system feel less safe and secure for participants). Beyond these guidelines the sessions are unstructured and open insuring that participants can talk about various issues based on their individual preferences.

**TAU control group**. Participants in this group continued their regular activities in their rehabilitation outpatient units without the addition of SCIT or TAFT. Study participants of all three groups all consumed a range of the same services which included psychiatric rehabilitation services in the community which provided support in key domains such as housing, work and leisure. The staff in the participating centers included a psychiatrist, who prescribed medication, and practitioners with various degrees of education and training from a broad range of professions (psychology, social work, nursing, occupational therapists, and paraprofessionals).

### Statistical Analysis

Analyses were computed using Predictive Analytics SoftWare (PASW, Version 25.0). To test whether the groups differed in their baseline scores on the scales and whether there were differences between participants who did and did not complete Time 2 assessments, two-way ANOVAs were performed. To examine improvement in the outcomes (SQoL, FEIT, Faux Pas task, SSPA, and AIHQ), mixed repeated measures ANOVAs were used with time (pre, post) and group (SCIT, TAFT, and TAU) as factors. A mixed repeated-measures ANOVA allows for an examination of the extent to which participants improved over time regardless of group, as well as whether they improved significantly more over time in one group versus the other (group–time interaction). To assess the overall effect sizes, partial eta squared (*η_p_^2^*) was computed with *η_p_^2^* = .01 indicating small, *η_p_^2^* = .059 medium, and *η_p_^2^* = .138 large effects (37). In case of a significant effect, a paired two-sample *t* test was conducted for each group comparing pre- and post-results. Effect sizes of paired tests were reported with Cohen’s *d* (37) with *d* = .2, indicating small; *d* = .5, medium; and *d* = .8 large effects. To examine whether the therapeutic alliance was associated with the change in the research variables, we performed a multiple regression, with the difference in each variable (difference between time 1 and time 2) as the dependent variable, and group (0-SCIT, 1-TAFT) and the four dimensions of the therapeutic as the independent variables.

## Results

Baseline scores of the research variables are presented in **Table 2**. As can be seen, analyses revealed no significant differences between the groups in any of the baseline assessments excluding the AIHQ—Anger score. The participants from the treatment groups (SCIT and TAFT) had higher AIHQ—Anger scores than did participants from the TAU group. In addition, there were significant differences in all of the subscales of the AIHQ (hostility bias, intentionality, anger, and blame) between those who did complete and those who did not complete post-intervention assessments. Those who completed post-intervention assessments had higher AIHQ scores at baseline. This effect was not group specific and no significant interaction effect between “group” and “attendance” in any of the variables.

**Table 2:** Means and Standard Deviations of the baseline scores on the scales and two-way ANOVA results for effects of “Group,” “Attendance,” and Interaction.

	SCIT *N*=62		TAFT *N*=58		TAU *N*=38		Group			Attendance			Interaction		
	Completers *N*=23 M (SD)	Non completers *N*=39 *M (SD)*	Completers *N*=20 M (SD)	Non completers *N*=38 *M (SD)*	Completers *N*=20 M (SD)	Non completers *N*=18 *M (SD)*	*F*	*P*	*η_p_^2^* **	*F*	*P*	*η_p_^2^* **	*F*	*P*	*η_p_^2^*
Social Quality of Life^a^	4.48 (1.22)	4.43 (1.54)	5.22 (1.35)	4.70 (1.46)	4.78 (1.40)	5.24 (1.29)	2.43	.091	.031	0.02	.876	.000	1.31	.274	.017
FEIT^b^	12.83 (2.86)	12.31 (2.45)	12.42 (3.52)	11.32 (3.02)	11.55 (3.20)	12.00 (3.18)	1.09	.339	.014	0.61	.434	.004	0.74	.477	.010
Faux Pas task^c^	30.79 (8.19)	31.44 (8.06)	30.11 (6.84)	28.28 (7.42)	27.95 (8.11)	31.00 (9.30)	0.87	.419	.012	0.21	.650	.001	0.97	.380	.013
SSPA^d^	47.13 (13.3)	52.54 (15.5)	48.90 (13.4)	47.08 (14.4)	54.00 (16.0)	50.88 (16.5)	0.95	.391	.013	0.00	.951	.000	1.21	.302	.016
AIHQ - Hostility Bias^e^	11.25 (3.73)	10.56 (3.12)	12.62 (4.20)	10.83 (4.05)	11.50 (3.95)	9.77 (3.51)	1.02	.362	.014	**4.73**	**.031**	**.033**	0.36	.701	.005
AIHQ - Intentionality score^e^	12.65 (3.31)	10.87 (3.16)	12.58 (4.66)	11.31 (3.27)	12.42 (3.76)	10.31 (2.80)	0.29	.750	.004	**8.22**	**.005**	**.054**	0.16	.853	.002
AIHQ – Anger score^e^	15.18 (4.31)	13.15 (4.89)	14.68 (5.52)	12.26 (4.73)	12.66 (4.50)	10.42 (4.01)	**3.39**	**.036**	**.045**	**7.51**	**.007**	**.050**	0.02	.977	.000
AIHQ - Blame score^e^	14.09 (4.09)	12.44 (4.52)	14.16 (5.92)	12.00 (4.95)	13.05 (4.50)	9.80 (3.82)	1.79	.171	.024	**8.32**	**.005**	**.055**	0.30	.738	.004

a Possible scores for the Social Quality of Life (SQoL) range from 1 to 7, with higher scores indicating better quality of life.

b FEIT, Face Emotion Identification Task. Possible scores for the FEIT range from 0 to 19 with higher scores indicating better emotion recognition.

c Possible scores for the FAUX PAS range from 0 to 36, with higher scores indicating better theory of mind (ToM).

d SSPA, Social Skill Performance Assessment. Possible scores for the SSPA range from 17 to 85 with higher scores indicating better social skills.

e AIHQ, Ambiguous Intentions Hostility Questionnaire. Possible scores for the AIHQ range from 5 to 25, with higher scores indicating greater bias.

**Figure 2:**
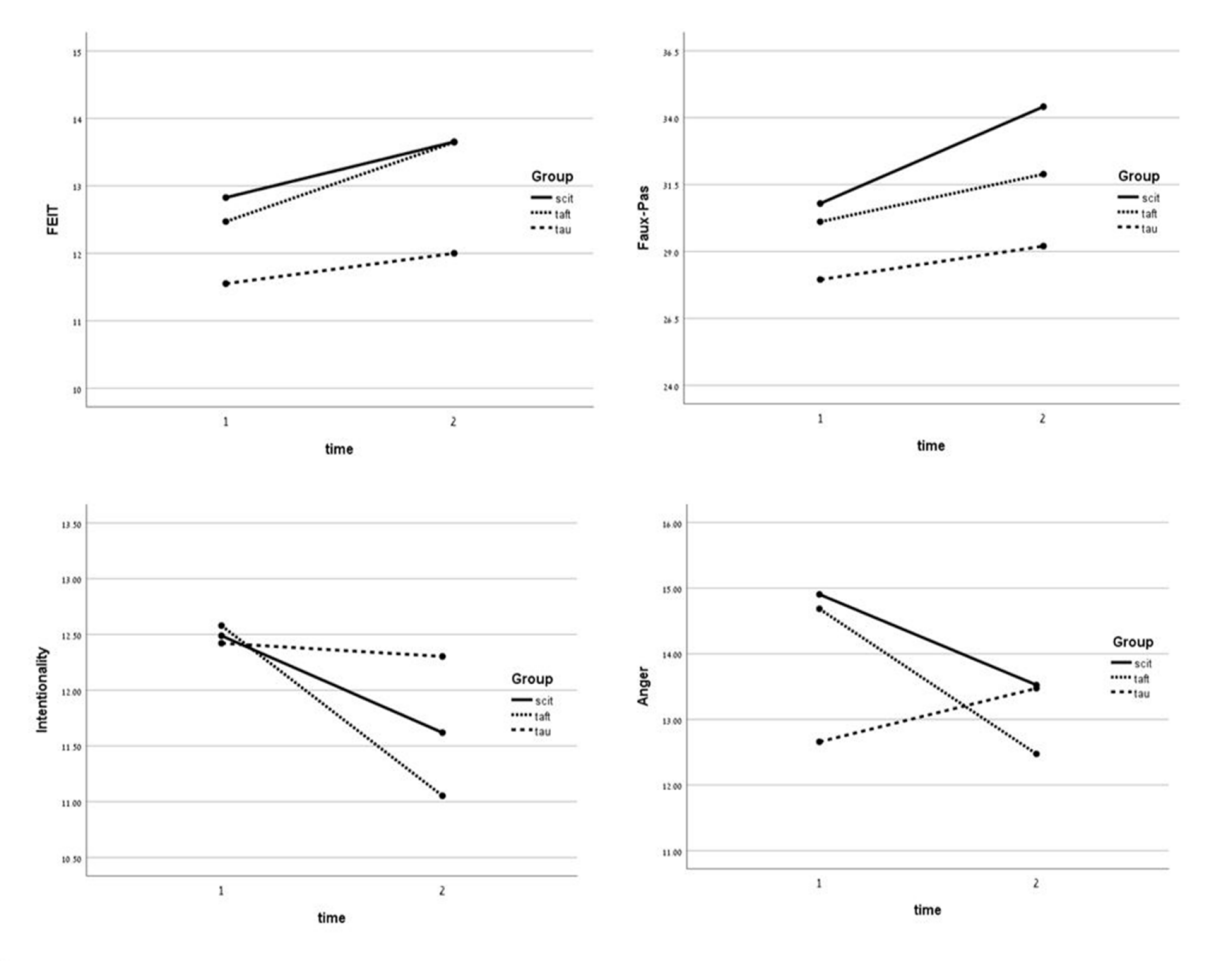
Means of the outcome measures for the 3 groups at the 2 assessments.

Repeated-measures ANOVAs were conducted with group as the independent variable and assessment point (pre-treatment vs. post-treatment) as time. An analysis was performed for each of the outcome measures. As can be seen in **Table 3**, there was a significant main effect for time, *F*(1,57) = 7.29, *p* < .01, *η_p_^2^* = .11, for the FEIT score. Although the interaction effect was not found to be significant, there was a significant difference in the mean change in the TAFT group, *t*(16) = -2.22, *p* < .05, *d* = -.54, and a not significant change in SCIT and TAU groups (see **Figure 2**). On the faux pas task, analyses revealed a significant main effect for time, *F*(1,57) = 5.99, *p* < .05, *η_p_^2^* = .10. Although the interaction effect was not found to be significant, there was a significant difference in the mean change in the SCIT group, *t*(21) = -2.58, *p* < .05, *d* = -.55, and a not significant change in TAFT and TAU groups (see **Figure 2**). For the AIHQ—Intentionality, analyses revealed a significant main effect for time, *F*(1,56) = 5.39, *p* < .05, *η_p_^2^* = .09. Although the interaction effect was not found to be significant, there was a significant difference in the mean change in the TAFT group, *t*(20) = 3.07, *p* < .01, *d* = .70, and a not significant change in SCIT and TAU groups (see **Figure 2**). Finally, a significant group x time interaction for the AIHQ—Anger was found, *F*(2,56) = 3.23, *p* < .05, *η_p_^2^* = .10. There was a significant difference in the mean change in the SCIT group, *t*(20) = 2.29, *p* < .05, *d* = .50, and in the TAFT group, *t*(18) = 2.75, *p* < .05, *d* = .63, in contrast to the TAU group, where there was not a significant difference (see **Figure 2**).

**Table 3:** Repeated measures ANOVAs for the outcome measures at pre-treatment and post-treatment for the three groups.

	SCIT *N* = 23		TAFT *N* = 20		TAU *N* = 20		Time		Group		Time×Group	
	Pre *M (SD)*	Post *M (SD)*	Pre *M (SD)*	Post *M (SD)*	Pre *M (SD)*	Post *M (SD)*	*P*	*η_p_^2^*	*P*	*η_p_^2^*	*P*	*η_p_^2^*
Social Quality of Life^a^	4.48 (1.22)	4.61 (1.31)	5.22 (1.35)	5.23 (1.53)	4.78 (1.40)	4.77 (1.32)	.701	.003	.222	.050	.869	.005
FEIT^b^	12.83 (2.86)	13.65 (2.71)	12.47 (3.71)	13.65 (3.45)	11.55 (3.20)	12.00 (3.73)	**.009**	**.113**	.254	.047	.640	.016
Faux Pas task^c^	30.79 (8.19)	34.41 (5.51)	30.11 (6.84)	31.89 (7.98)	27.95 (8.11)	29.20 (8.51)	**.017**	**.095**	.160	.062	.517	.023
SSPA^d^	47.13 (13.3)	52.39 (16.6)	48.90 (13.4)	50.20 (12.7)	54.00 (16.0)	49.70 (16.2)	.728	.002	.800	.007	.194	.053
AIHQ - Hostility Bias^e^	10.93 (3.50)	10.19 (2.34)	12.63 (4.20)	11.64 (4.50)	11.64 (3.70)	12.06 (3.61)	.298	.020	.290	.045	.358	.037
AIHQ - Intentionality score^e^	12.49 (3.31)	11.62 (2.09)	12.58 (4.66)	11.05 (4.78)	12.42 (3.76)	12.30 (2.96)	**.024**	**.088**	.882	.004	.300	.042
AIHQ – Anger score^e^	14.90 (4.21)	13.53 (3.39)	14.68 (5.52)	12.47 (5.64)	12.66 (4.50)	13.47 (5.26)	.067	.059	.711	.012	**.047**	**.103**
AIHQ - Blame score^e^	14.29 (4.09)	13.00 (3.61)	14.16 (5.92)	13.00 (5.73)	13.05 (4.50)	13.63 (4.42)	.166	.034	.975	.001	.171	.061

a Possible scores for the Social Quality of Life (SQoL) range from 1 to 7, with higher scores indicating better quality of life.

b FEIT, Face Emotion Identification Task. Possible scores for the FEIT range from 0 to 19 with higher scores indicating better emotion recognition.

c Possible scores for the FAUX PAS range from 0 to 36, with higher scores indicating better theory of mind (ToM).

d SSPA, Social Skill Performance Assessment. Possible scores for the SSPA range from 17 to 85 with higher scores indicating better social skills.

e AIHQ, Ambiguous Intentions Hostility Questionnaire. Possible scores for the AIHQ range from 5 to 25, with higher scores indicating greater bias.

Another repeated-measures ANOVA was conducted with group as the independent variable and three assessment points (pre, post, and follow-up) as time. This analysis was conducted on only 43 subjects (17 from the SCIT group, 15 from the TAFT group and 11 from the TAU group). Analyses revealed a significant main effect for time, *F*(2,72) = 7.69, *p* < .01, *η_p_^2^* = .18, for the faux pas score. In an analysis performed on each group separately, a significant main effect for time was found only in the SCIT group, *F*(2,30) = 4.87, *p* < .05, *η_p_^2^* = .25, and only between the second (post) and third (follow-up) assessment. In addition, there was a significant main effect for time, *F*(2,72) = 7.69, *p* < .01, *η_p_^2^* = .18, for the SSPA score. The improvement was significant only in the TAFT group, *F*(2,28) = 4.12, *p* < .05, *η_p_^2^* = .23, and only between the second (post) and third (follow-up) assessment. Finally, there was a significant main effect for time, *F*(2,72) = 7.69, *p* < .01, *η_p_^2^* = .18, for the AIHQ—Intentionality score. The improvement was significant only in the TAFT group, *F*(2,26) = 5.55, *p* < .01, *η_p_^2^* = .30, and only between the first (pre) and second (post) assessment. No significant main effect for group or for the group × time interaction was found in any of the variables.

To examine whether the therapeutic alliance was associated with the changes in the research variables, we performed a multiple regression, with the difference in each variable (difference between time 1 and time 2) as the dependent variable, and group (0-SCIT, 1-TAFT) and the four dimensions of the therapeutic alliance (safety within the therapeutic situation, shared sense of purpose, engagement in the therapeutic process, and emotional connection to the therapist) as the independent variables. We found that “engagement in the therapeutic process” was a significant predictor of change in SQoL (β = .61, p < .05). A higher score on “engagement in the therapeutic process” was associated with a larger change in SQoL. In addition, “shared sense of purpose” was a significant predictor of change in the faux pas task (β = .52, p < .05). A higher score on “shared sense of purpose” was associated with a greater change in the faux pas task.

To examine fidelity effects, scores of fidelity were computed for each group. The majority of the groups (four SCIT groups and four TAFT groups) showed high fidelity, with only one SCIT group and one TAFT group (each included only two participants) showing low fidelity. A re-examination of the findings excluding these participants did not reveal additional new findings.

## Discussion

The current study compared the effectiveness of a didactic approach for improving social functioning by means of improving social cognition (SCIT) to an experiential approach for improving social functioning through creating a positive group therapeutic alliance (TAFT). Results showed a relatively low rate of completion in both groups, with improvement in only a few of the outcome variables in both groups. Specifically, participants in the TAFT group showed an improvement in emotion identification and in intentionality bias, and participants in the SCIT group showed improvement in TOM. In addition, participants in both SCIT and TAFT showed an improvement in their anger bias scores in comparison to participants in TAU. Changes following the interventions were related to better therapeutic alliance. At follow-up, participants in the SCIT intervention showed improvement in TOM and participants in the TAFT intervention showed improvement in social functioning and intentionality bias. No additional benefits were uncovered following the interventions.

These findings are largely consistent with the contextual model of psychotherapy (38), supporting the classic concept of the “Dodo effect” (39); that is, all types of interventions are equally beneficial as they all share common factors. This model views the changes that can occur in psychotherapy as being attributed to three pathways: the real relationship between therapist and client within the special framework of therapeutic relations (e.g., confidentiality), the expectations one has from the intervention, and specific therapeutic actions. When referring to specific therapeutic actions, the contextual model does not mean that therapeutic actions of one specific approach are superior to those of another. Rather, it means that different therapeutic actions (in the current study, CBT techniques or reflecting on and improving interpersonal relations in the group) that induce healthy behavior are responsible for change. Notably, as emphasized by Wampold (38), therapeutic alliance must be established for the three pathways to be facilitated.

Although participants in each of the groups (SCIT and TAFT) did not show improvement in the same social cognition outcomes (both post intervention and follow-up), they showed better results than participants in the TAU group. That is, participation in each of the two interventions resulted in more benefits than TAU, suggesting that both a dyadic training approach as well as a more dynamic experiential approach enable changes in social outcome. Whereas in SCIT participants used specific exercises aimed to enhance social outcomes, in TAFT, participants practiced their social abilities aimed towards building a therapeutic alliance. Importantly, the two approaches differ with regard to their emphasis on illness and deficits; that is, the SCIT intervention is deficit-focused whereas the TAFT is not. Whether the TAFT group includes discussion about the illness and related deficits depends only on whether it is raised by the group members.

Of note are the high dropout rates. Although the dropout rates were similar to attrition rates reported in most studies [see reviewed studies in Ref. (40)], this factor limits the power of the study. However, it is worth considering that when someone stops attending an intervention, it does not necessarily mean he or she has “dropped out.” Rather, it could be that people often choose to discontinue an intervention for reasons other than being unsatisfied from or feeling they did not benefit from the sessions they did attend (41, 42).

With regard to the limited effects of the interventions, a recent meta-analysis showed that among most people with SMI, metacognitive approaches are superior to other psychological treatments, such as cognitive remediation and CBT (43). This finding is in line with updated research showing that metacognition and symptoms are more central than social cognition in a network of cognition, metacognition, social cognition, and symptoms (44). It is also in accord with a recent review that differentiated between metacognitive approaches and other psychological approaches in the treatment of psychosis and suggested that the metacognitive approaches are unique in their emphasis on sense-making rather than skill learning (45). Clinical implications of the current study suggest that the use of common factors, especially therapeutic alliance, should be enhanced. This suggestion is in line with a recent systematic review on therapeutic alliance among persons with psychosis, showing the important role it plays in both reducing symptoms, as well as in enhancing psychological outcomes, such as self-esteem (24). In addition, tailoring the contextual factors (e.g., size of groups, length of sessions) to clients may increase adherence and facilitate outcome. Of note, as one of the major concerns of persons with SMI is the meeting of their social needs (5), it is important to address these needs with either a more structured approach such as the SCIT intervention or with a more open-dynamic approach such as the TAFT intervention.

With these implications in mind, limitations of the current study should be taken into account. As mentioned above, the high number of participants who discontinued may have limited the study’s power. In addition, the sample was heterogeneous, and conclusions regarding the benefits of the interventions for people with specific diagnoses cannot be inferred. Relatedly, the lack of a specific SMI diagnosis, due to the sample being community-based and characterized not by a specific diagnosis but rather by the utilization of psychiatric rehabilitation services, is a further limitation. Future studies that address these issues, as well as the ideas discussed above, including an assessment of people who discontinue participation in interventions, are needed to further validate the study findings and explore best practice for persons with SMI.

## Data Availability Statement

The datasets generated for this study are available on request to the corresponding author.

## Ethics Statement

This study was carried out in accordance with the recommendations of guidelines of the ethical committee of Bar-Ilan University with written informed consent from all subjects. All subjects gave written informed consent in accordance with the Declaration of Helsinki. The protocol was approved by the ethical committee of the Department of Psychology at Bar-Ilan University, as well as by the ethical committee of the psychiatric hospital Abarbanel. Note that this hospital did not take part in the trial.

## Author Contributions

IHO and DR designed the study and were the directors of the project. ALR collected the data and coordinated the implementation of interventions. MME was in charge of analyses of findings. All authors contributed to interpretation of findings and writing of the manuscript.

## Funding

This study was funded by a grant from the Israel Science Foundation (grant 329/13) of authors IHO and DR.

## Conflict of Interest Statement

The authors declare that the research was conducted in the absence of any commercial or financial relationships that could be construed as a potential conflict of interest.

## References

[1] Kurtz MM Richardson CL Social cognitive training for schizophrenia: a meta-analytic investigation of controlled research. Schizophr Bull (2011) 38(5):1092–104. 10.1093/schbul/sbr036 PMC3446217 21525166

[2] Couture SM Penn DL Roberts DL The functional significance of social cognition in schizophrenia: a review. Schizophr Bull (2006) 32(suppl_1):S44–S63. 10.1093/schbul/sbl029 16916889 PMC2632537

[3] Brüne M Abdel-Hamid M Lehmkämper C Sonntag C Mental state attribution, neurocognitive functioning, and psychopathology: what predicts poor social competence in schizophrenia best? Schizophr Res (2007) 92(1–3):151–9. 10.1016/j.schres.2007.01.006 17346931

[4] Fernández-Sotos P Navarro E Torio I Dompablo M Fernández-Caballero A Rodriguez-Jimenez R Pharmacological interventions in social cognition deficits: a systematic mapping review. Psychiatry Res (2018) 270:57–67. 10.1016/j.psychres.2018.09.012 30245378

[5] Middelboe T Mackeprang T Hansson L Werdelin G Karlsson H Bjarnason O The Nordic Study on schizophrenic patients living in the community. Eur Psychiatry (2001) 16(4):207–14. 10.1016/S0924-9338(01)00566-1 11418270

[6] Roberts DL Penn DL Combs DR Social cognition and interaction training (SCIT): group psychotherapy for schizophrenia and other psychotic disorders, clinician guide. New York: Oxford University Press (2015). 10.1093/med:psych/9780199346622.001.0001

[7] Combs DR Adams SD Penn DL Roberts D Tiegreen J Stem P Social Cognition and Interaction Training (SCIT) for inpatients with schizophrenia spectrum disorders: preliminary findings. Schizophr Res (2007a) 91(1–3):112–6. 10.1016/j.schres.2006.12.010 17293083

[8] Combs DR Penn DL Tiegreen JA Nelson A Ledet SN Basso MR Stability and generalization of social cognition and interaction training (SCIT) for schizophrenia: six-month followup results. Schizophr Res (2009) 112:196–7. 10.1016/j.schres.2009.04.010 19411160

[9] Roberts DL Penn DL Social cognition and interaction training (SCIT) for outpatients with schizophrenia: a preliminary study. Psychiatry Res (2009) 166(2–3):141–7. 10.1016/j.psychres.2008.02.007 19272654

[10] Roberts DL Penn DL Labate D Margolis SA Sterne A Transportability and feasibility of social cognition and interaction training (SCIT) in community settings. Behav Cogn Psychother (2010) 38(1):35–47. 10.1017/S1352465809990464 19857363

[11] Iyer SN Rothmann TL Vogler JE Spaulding WD Evaluating outcomes of rehabilitation for severe mental illness. Rehabil Psychol (2005) 50(1):43. 10.1037/0090-5550.50.1.43

[12] Kessler RC Barker PR Colpe LJ Epstein JF Gfroerer JC Hiripi E Screening for serious mental illness in the general population. Arch Gen Psychiatry (2003) 60(2):184–9. 10.1001/archpsyc.60.2.184 12578436

[13] Chan RC Gao XJ Li XY Li HH Cui JF Deng YY The Social Cognition and Interaction Training (SCIT): an extension to individuals with schizotypal personality features. Psychiatry Res (2010) 178(1):208–10. 10.1016/j.psychres.2010.03.017 20421136

[14] Penn D Roberts DL Munt ED Silverstein E Jones N Sheitman B A pilot study of social cognition and interaction training (SCIT) for schizophrenia. Schizophr Res (2005) 80(2–3):357–9. 10.1016/j.schres.2005.07.011 16139480

[15] Hasson-Ohayon I Mashiach–Eizenberg M Avidan M Roberts D Roe D Social cognition and interaction training: preliminary results of a RCT study in a community setting in Israel. Psychiatr Serv (2014) 65(4):555–8. 10.1176/appi.ps.201300146 24687108

[16] Lahera G Benito A Montes JM Fernandez-Liria A Olbert CM Penn DL Social cognition and interaction training (SCIT) for outpatients with bipolar disorder. J Affect Disord (2013) 146(1):132–6. 10.1016/j.jad.2012.06.032 22840617

[17] Turner-Brown LM Perry TD Dichter GS Bodfish JW Penn DL Brief report: feasibility of social cognition and interaction training for adults with high functioning autism. J Autism Dev Disord (2008) 38(9):1777–84. 10.1007/s10803-008-0545-y PMC2646378 18246419

[18] Friedlander ML Escudero V Heatherington L Therapeutic alliances in couple and family therapy: an empirically informed guide to practice. Washington, DC: American Psychological Association (2006). 10.1037/11410-000

[19] Levy-Frank I Hasson-Ohayon I Kravetz S Roe D Family psychoeducation and therapeutic alliance focused interventions for parents of a daughter or son with a severe mental illness. Psychiatry Res (2011) 189(2):173–9. 10.1016/j.psychres.2011.02.012 21482437

[20] Levy-Frank I Hasson-Ohayon I Kravetz S Roe D A narrative evaluation of a psychoeducation and a therapeutic alliance intervention for parents of persons with a severe mental illness. Fam Process (2012) 51(2):265–80. 10.1111/j.1545-5300.2012.01398.x 22690865

[21] Penn DL Mueser KT Tarrier N Gloege A Cather C Serrano D Supportive therapy for schizophrenia: possible mechanisms and implications for adjunctive psychosocial treatments. Schizophr Bull (2004) 30(1):101–12. 10.1093/oxfordjournals.schbul.a007055 15176765

[22] Goldsmith LP Lewis SW Dunn G Bentall RP Psychological treatments for early psychosis can be beneficial or harmful, depending on the therapeutic alliance: an instrumental variable analysis. Psychol Med (2015) 45(11):2365–73. 10.1017/S003329171500032X PMC4501302 25805118

[23] Lecomte T Laferrière-Simard M-C Leclerc C What does the alliance predict in group interventions for early psychosis? J Contemp Psychother (2012) 42(2):55–61. 10.1007/s10879-011-9184-2

[24] Shattock L Berry K Degnan A Edge D Therapeutic alliance in psychological therapy for people with schizophrenia and related psychoses: a systematic review. Clin Psychol Psychother (2018) 25(1):e60–e85. 10.1002/cpp.2135 28961352

[25] Hasson-Ohayon I Goldzweig G Arnon-Ribenfeld N Mashiach-Eizenberg M Kravetz S Roe D The use of the social skills performance assessment (SSPA) among persons with serious mental illness: psychometric assessment and network analysis. J Ment Health (2018a) 21:1–8. 10.1080/09638237.2018.1521934 30463463

[26] Hasson-Ohayon I Mashiach-Eizenberg M Arnon-Ribenfeld N Kravetz S Roe D Neuro-cognition and social cognition elements of social functioning and social quality of life. Psychiatry Res (2017) 258:538–43. 10.1016/j.psychres.2017.09.004 28916297

[27] Struch N Shereshevsky Y Naon D Daniel D Fischman N People with severe mental disorders in Israel: an integrated view of the service systems. Jerusalem, Israel: The Myers-JDC-Brookdale Institute (2009).

[28] Kerr SL Neale JM Emotion perception in schizophrenia: specific deficit or further evidence of generalized poor performance? J Abnorm Psychol (1993) 102(2):312. 10.1037//0021-843X.102.2.312 8315144

[29] Penn DL Combs DR Ritchie M Francis J Cassisi J Morris S Emotion recognition in schizophrenia: further investigation of generalized versus specific deficit models. J Abnorm Psychol (2000) 109(3):512. 10.1037//0021-843X.109.3.512 11016120

[30] Shamay-Tsoory SG Tomer R Berger BD Goldsher D Aharon-Peretz J Impaired “affective theory of mind” is associated with right ventromedial prefrontal damage. Cogn Behav Neurol (2005) 18(1):55–67. 10.1097/01.wnn.0000152228.90129.99 15761277

[31] Stone VE Baron-Cohen S Knight RT Frontal lobe contributions to theory of mind. J Cogn Neurosci (1998) 10:640–56. 10.1162/089892998562942 9802997

[32] Combs DR Penn DL Wicher M Waldheter E The Ambiguous Intentions Hostility Questionnaire (AIHQ): a new measure for evaluating hostile social-cognitive biases in paranoia. Cogn Neuropsychiatry (2007b) 12(2):128–43. 10.1080/13546800600787854 17453895

[33] Patterson TL Moscona S McKibbin CL Davidson K Jeste DV Social skills performance assessment among older patients with schizophrenia. Schizophr Res (2001) 48(2–3):351–60. 10.1016/S0920-9964(00)00109-2 11295387

[34] Becker M Diamond R Sainfort F A new patient focused index for measuring quality of life in persons with severe and persistent mental illness. Qual Life Res (1993) 2(4):239–51. 10.1007/BF00434796 8220359

[35] Kravetz S Faust M Dasberg I A comparison of care consumer and care provider perspectives on the quality of life of persons with persistent and severe psychiatric disabilities. Psychiatr Rehabil J (2002) 25(4):388. 10.1037/h0094998 12013267

[36] Van Nieuwenhuizen C Schene AH Boevink WA Wolf J Measuring the quality of life of clients with severe mental illness: a review of instruments. Psychiatr Rehabil J (1997) 20:33–42.

[37] Cohen J Statistical power analysis for the behavioral sciences. 2nd ed Hillsdale, NJ: Erlbaum (1988).

[38] Wampold BE Imel ZE The great psychotherapy debate: the evidence for what makes psychotherapy work. New York: Routledge (2015). 10.4324/9780203582015

[39] Rosenzweig S Some implicit common factors in diverse methods of psychotherapy. Am J Orthopsychiatry (1936) 6:412–5.

[40] Barrett MS Chua WJ Crits-Christoph P Gibbons MB Thompson D Early withdrawal from mental health treatment: implications for psychotherapy practice. Psychotherapy (2008) 45(2):247. 10.1037/0033-3204.45.2.247 19838318 PMC2762228

[41] Roe D Hasson-Ohayon I Gornemann MI Reasons for discontinuing attendance at a group intervention among people with serious mental illness. Psychiatr Serv (2016) 67(9):1043–1043. 10.1176/appi.ps.201600172 27582352

[42] Roe D Davidson L Noncompliance, nonadherence, and dropout: outmoded terms for modern recovery-oriented mental health. Psychiatr Serv 68(10):1076–8. 10.1176/appi.ps.201600522 28669286

[43] Philipp R Kriston L Lanio J Kühne F Härter M Moritz S Effectiveness of metacognitive interventions for mental disorders in adults—a systematic review and meta-analysis (METACOG). Clin Psychol Psychother (2018) 26(2):227–40. 10.1002/cpp.2345 30456821

[44] Hasson-Ohayon I Goldzweig G Lavi-Rotenberg A Luther L Lysaker PH The centrality of cognitive symptoms and metacognition within the interacting network of symptoms, neurocognition, social cognition and metacognition in schizophrenia. Schizophr Res (2018b) 202:260–6. 10.1016/j.schres.2018.07.007 30001972

[45] Lysaker PH Gagen E Moritz S Schweitzer RD Metacognitive approaches to the treatment of psychosis: a comparison of four approaches. Psychol Res Behav Manag (2018) 11:341–51. 10.2147/PRBM.S146446 PMC6130286 30233262

